# Schools for Recent Graduates

**Published:** 1906-09-01

**Authors:** 


### Schools for Recent Graduates.

### IN LONDON.

#### The London School of Clinical Medicine, Seamen's Hospital ("Dreadnought"), Greenwich.

The practice of the hospital, and of the dispensaries, is reserved entirely for qualified medical practitioners.

The hospital is easily reached from London: By train from Charing Cross, Waterloo Junction, Cannon Street, or London Bridge, to Greenwich Station, which is within five minutes of the hospital; or from Victoria, Holborn Viaduct, St. Paul's to Greenwich Park Station; or by tram from the south side of the Bridges. The train takes from 20 to 25 minutes, and the tram about 40 minutes. There are special departments for study of eye, throat, nose, and ear, skin, teeth, and radiography. Practical classes are arranged on all necessary subjects, provided not less than six students enter.

The supply of material affords exceptional facilities for practical instruction in operative surgery, and in pathology on the cadaver.

Arrangements have been made with the Royal Waterloo Hospital for graduates who desire instruction in diseases of women and children, with the Bethlem Hospital for instruction in mental diseases, and with the General Lying-in Hospital for the prosecution of study in midwifery.

The school supplies opportunities for professional tuition to medical officers of the public services, and its certificates for attendance during study-leave are accepted by the Admiralty and the War Office. For general practitioners who desire from time to time to bring themselves into touch with the most recent scientific advances, as well as for graduates who are reading for the higher qualifications, there is ample provision for the acquisition of practical knowledge and technical skill in every department of medicine and surgery.

For attendance on the ordinary practice of the hospital in the wards, operating theatres, post-mortem rooms, and out-patient rooms, including admission to ward cliniques and clinical lectures:—For one month, £3 3s.; for each month subsequent to the first, £1 1s.; for each practical class during the session, £2 2s.; except operative surgery—minor course (4 men), £4 4s.—major course (2 men), £7 7s.; gynæcology, £3 3s.; clinical pathology, microscopy, and bacteriology, £3 3s.

A composite fee covering admission to all the practical classes except operative surgery, as well as to the general practice of the hospital during a complete session, £16 16s.

Fee for vacation course in September, including one month's hospital practice, also attendance on lectures and demonstrations, £5 5s.

### The West London Hospital and Post-Graduate College.

The West London Hospital is a fully equipped general hospital having 159 beds and an out-patient department at which some 30,000 new patients are seen every year. In addition to the general physicians and general surgeons the staff includes representatives of all branches of special practice, each of whom conducts an appropriate clinical service at the hospital. The whole of this practice, both indoor and outdoor, is utilised for purposes of clinical teaching. The college in connection with the hospital has been gradually extended and developed so that it may now claim to be a thoroughly well organised and well-equipped institution; recently there has been added a new pathological laboratory, and the arrangements for teaching microscopy, bacteriology, and Tropical medicine have been brought well up-to-date. Lectures on medicine, surgery, etc., are given daily by members of the staff, and, for those who desire it, special instruction is provided with a view to prepare candidates for the higher examinations. There is ample provision for the convenience of practitioners attending the College in the shape of reading-room, smoking-room, library, etc., and though Hammersmith, where the hospital is situated, is not exactly in the centre of London, the facilities for getting there will satisfy the most exacting. The fees for hospital practice, including all the ordinary demonstrations and lectures, are £2 2s. for one month, £5 5s. for three months, £8 8s. for six months, and £12 12s. for 12 months. To put the position in brief, it is certain that for these fees the practitioner has every chance of following under experienced teachers the practice of a fairly large modern hospital. The winter session commences on Monday, October 8. Any further information can be obtained on application to the Dean, Mr. L. A. Bidwell.

### The Medical Graduates' College and Polyclinic.

This institution offers many advantages for very modest fees. There is a special clinical service on five days of the week at 4 p.m., when selected cases are exhibited and made the text for a clinical commentary and discussion; and at 5.15 p.m., on four days of the week, there is also a clinical lecture or lantern demonstration. Both clinics and lectures are conducted by physicians and surgeons from the metropolitan schools and hospitals, so that they are often of special interest and value. The opportunities for seeing clinical cases and gathering the teaching they convey are, therefore, excellent. The fee for admission to this part of the work is £1 1s., and this includes the use of the museum, reading-room, etc. There are also practical classes for instruction in the use of the ophthalmoscope and laryngoscope, the estimation of refraction and the examination of cases of nervous disease, and in gynæcology, otology, bacteriology, etc. Special tutorial classes meet the needs of practitioners preparing for the higher examinations in medicine and surgery. A prospectus, with particulars of fees, etc., can be obtained from the Medical Superintendent, the Polyclinic, 22 Chenies Street, Gower Street, W.C.

### The London Post-graduate Association.

In addition, the "London Post-Graduate Association" issues tickets which admit to the clinical practice of a number of the leading general and special hospitals in the metropolis. The fee for a term of three months is £10 10s. Applications should be addressed to the Secretary, London Post-Graduate Association, Examination Hall, Victoria Embankment, W.C.

### North-East London Post-Graduate College.

This school is in connection with the Tottenham Hospital, N., recognised by the University of London as a place of post-graduate study. Opportunities are here afforded for attending demonstrations of various branches of medicine, surgery, and gynæcology, with opportunities for clinical instruction in diseases of the eye, ear, throat, nose, skin, and in fevers, diseases of children, psychological medicine, anæsthetics, and dentistry. Special classes with a limited attendance have been arranged for these subjects. The fee for a three months' course in any single department is one guinea, or three guineas admits for a similar term to the whole practice of the hospital. A perpetual ticket costs five guineas. Additional information, with syllabus of lectures and special classes, from the Dean, Dr. T. T. Whiting, 142 Harley Street, W.

### General Hospitals.

University College has arrangements by which Dr. Vaughan Harley holds classes in practical pathological chemistry for the advantage of qualified practitioners and advanced students. Each student not only does the ordinary class analysis, but learns to apply the results to practice. The course includes analysis of saliva, stomach contents, fæces, urine calculi, and pathological fluids. The laboratory is open from 9 A.M. to 5 P.M. The fee for the class, including apparatus and material, is £5 5s. Charing Cross Hospital arranges a special series of lectures and practical demonstrations during the year, each course consisting of ten meetings and lasting ten weeks. The class meets each Thursday in the board room of the Hospital, and thence proceeds to the department in which the demonstration may be held. Apply to Dr. Willcocks at the Hospital.

### Cambridge.

Cambridge admits as advanced students graduates of other universities, and grants certificates of research after a residence of one academical year. After two years' residence the student is admissible to the degree of B.A.

### IN SCOTLAND.

### Post-Graduate Teaching in Edinburgh.

The four Scottish Universities, at three of which are situated medical schools of no mean reputation, have been but litle distinguished in the past for their encouragement of post-graduate study.

St. Andrew's and Aberdeen concern themselves with the graduate no further than by offering him a degree in Public Health. Beyond this, Glasgow, despite the great wealth of clinical material possessed by that large city, offers courses only in practical bacteriology, pathological histology, physiological chemistry, embryology, and a few of the special departments—*e.g.* diseases of the nose and ear, diseases of the eye, etc.—which are attended usually by senior students.

### The Edinburgh Scheme.

In Edinburgh, however, a more elaborate scheme is now available for the graduate desirous of continuing his studies. The University of this city, in addition to teaching the subject of public health, has for some years provided advanced classes in the ordinary medical subjects for senior students and graduates, such as bacteriology, organic chemistry, and embryology, while numerous laboratories attached to the various professorial chairs have been open for research under certain restrictions. These laboratories are increasing yearly in equipment and efficiency, mainly as a result of the funds derived from Mr. Andrew Carnegie's gift of £2,000,000 to the Scottish Universities. For this purpose, too, a magnificent laboratory has been organised and equipped by the Royal College of Physicians of Edinburgh, where any medical graduate may immediately obtain permission to conduct scientific research and can have skilled advice and aid freely placed at his disposal. There has hitherto, however, been little organised attempt to provide that practical tuition by experts in special subjects, which forms so important a feature at the Continental medical schools, notably at Vienna. It is true that for about a quarter of a century the teachers of the Extra-Mural School, as distinguished from the University, have organised and taught classes in certain of the subjects upon which medical men after graduation desire special information or practical experience. These classes have included practical courses upon ophthalmoscopy, diseases of the blood, diseases of the chest, neurology, medical electricity, tropical diseases, practical gynæcology, and operative midwifery, conducted in each case by a man who was an expert upon the subject concerned. The courses have, in general, been held coincidently with the ordinary medical classes during the winter and summer sessions, though in the case of several the teachers are ready to conduct classes at any time when a sufficient number (generally twelve) members present themselves. The fee for each course, which lasts for from three to six weeks, has been two or three guineas.

### A Holiday Course.

This year, however, a scheme has taken shape, devised conjointly by the University and the Royal Colleges of Edinburgh, to provide courses which shall attract graduates who have recently obtained their diploma—medical officers home on furlough, practitioners resident in country districts, foreign doctors anxious to study English, and any others who may desire a brief *resumé* of the essentials and recent advances in various special subjects. Accordingly a holiday course has been drawn up, lasting from September 17 to October 6, and embracing lectures and demonstrations on some twenty or thirty subjects. The course has been arranged so that each member may attend a large number of subjects, for each of which a teacher has been chosen who is regarded as to some extent an expert in his subject. Most of the subjects are covered in three or six demonstrations of an hour or two hours each, and the fee charged is a composition one of six guineas for the whole course, or three guineas for any three subjects selected. Those attending the course may, if they so desire, obtain rooms in one of the University Halls at a charge of £1 10s. per week for board and lodging. Considerable efforts are being put forth to render the course successful in its social aspect, and there will be numerous entertainments and opportunities of visiting those points of beauty and of antiquarian interest with which the environs of Edinburgh abound.

### List of Subjects.

Among the subjects dealt with are the following: Anæsthetics, including demonstrations upon patients of the administration of nitrous oxide, ethyl chloride, ether, chloroform, etc.; bacteriology, in which the preparation of media, sterilisation, and the commoner pathogenic organisms are studied in six demonstrations; pathology of the eye in three demonstrations, and six demonstrations illustrated by cases of the varying normal and abnormal ophthalmoscopic appearances; examination of the cerebro-spinal fluid (two demonstrations); modern gastric methods (six demonstrations); practical examination of abnormal urines (three demonstrations); venereal diseases (three demonstrations). Besides these, various selected chapters will be demonstrated in museums or cliniques upon diseases of children, of the ear, nose, and throat, of the heart, of the lungs, of the nervous system, of the skin, and of the urinary tract; upon surgical pathology (three demonstrations), and upon pathological anatomy (three demonstrations). Some of the major gynæcological operations will be performed according as cases are available at the time, while various novel subjects are to be taken up in clinical medicine and clinical surgery.

The course in this its first session will be largely an experiment, but if it is well attended, not only is there an intention to increase the number of subjects taught in future years, but provision will be made, as in foreign schools, for holding more extended courses at irregular intervals during the year whenever sufficient numbers present themselves.

Glasgow Royal Infirmary.

The members of the staff of the hospital, with the hearty co-operation of the managers, have now arranged a third series of post-graduate classes. These are to be opened on September 4th by Major George Lamb, M.D., I.M.S. The subject of his address is the Etiology of Plague, one to which he has devoted much attention and original research. This year the staff of the Royal Infirmary have taken the matter up, and have organised a series of practical and clinical classes. The first and second series have already proved very successful, and have been well attended. The third course will include in all about one hundred and twenty meetings. Apply to Dr. Maxtone Thom, Superintendent of the Royal Infirmary.

---

IN IRELAND.

Trinity College, Dublin.

A three weeks' post graduates' course is given during the summer session. The fee is £5 5s. A limited number of the class can live in the college rooms and dine in hall, at a charge of £1 per week. Apply Dr. Alfred Parsons, Lower Fitzwilliam Street, Dublin. A school has been formed to prepare candidates for the Royal Navy, the Royal Army Medical Corps, and the Indian Medical Services. The classes are held twice a year. Apply Dr. C. A. K. Ball, Merrion Square, Dublin.

Belfast.

Special summer classes are formed in bacteriology, clinical pathology, neurology, chemical physiology; and during the winter facilities are afforded to graduates to work at these subjects.

